# Predictors of complications occurring after open and robot-assisted prostate cancer surgery: a retrospective evaluation of 1062 consecutive patients treated in a tertiary referral high volume center 

**DOI:** 10.1007/s11701-021-01192-w

**Published:** 2021-02-09

**Authors:** Antonio Benito Porcaro, Alessandro Tafuri, Riccardo Rizzetto, Nelia Amigoni, Marco Sebben, Aliasger Shakir, Katia Odorizzi, Alessandra Gozzo, Sebastian Gallina, Alberto Bianchi, Paola Irene Ornaghi, Stefano Zecchini Antoniolli, Vincenzo Lacola, Matteo Brunelli, Filippo Migliorini, Maria Angela Cerruto, Salvatore Siracusano, Walter Artibani, Alessandro Antonelli

**Affiliations:** 1 Department of Urology, University of Verona, Azienda Ospedaliera Universitaria Integrata Verona, Piazzale Stefani 1, 37126 Verona, Italy; 2 Department of Neuroscience, Imaging and Clinical Sciences, “G. D’Annunzio” University, Chieti-Pescara, Italy; 3 Sacro Cuore Don Calabria Hospital, IRCCS, Negrar, Italy; 4 USC Institute of Urology and Catherine and Joseph Aresty Department of Urology, Keck School of Medicine, University of Southern California (USC), Los Angeles, CA USA; 5 Department of Pathology, University of Verona, Azienda Ospedaliera Universitaria Integrata Verona, Verona, Italy

**Keywords:** Prostate cancer, Radical prostatectomy, Complications, Urological complications

## Abstract

To investigate factors associated with the risk of major complications after radical prostatectomy (RP) by the open (ORP) or robot-assisted (RARP) approach for prostate cancer (PCa) in a tertiary referral center. 1062 consecutive patients submitted to RP were prospectively collected. The following outcomes were addressed: (1) overall postoperative complications: subjects with Clavien-Dindo System (CD) one through five versus cases without any complication; (2) moderate to major postoperative complications: cases with CD < 2 vs.  ≥ 2, and 3) major post-operative complications: subjects with CDS CD ≥  3 vs.  < 3. The association of pre-operative and intra-operative factors with the risk of postoperative complications was assessed by the logistic regression model. Overall, complications occurred in 310 out of 1062 subjects (29.2%). Major complications occurred in 58 cases (5.5%). On multivariate analysis, major complications were predicted by PCa surgery and intraoperative estimated blood loss (EBL). ORP compared to RARP increased the risk of major CD complications from 2.8 to 19.3% (OR = 8283; *p* < 0.0001). Performing ePLND increased the risk of major complications from 2.4 to 7.4% (OR = 3090; *p* < 0.0001). Assessing intraoperative blood loss, the risk of major postoperative complications was increased by BL above the third quartile when compared to subjects with intraoperative blood loss up to the third quartile (10.2% vs. 4.6%; OR = 2239; 95%CI: 1233–4064). In the present cohort, radical prostatectomy showed major postoperative complications that were independently predicted by the open approach, extended lymph-node dissection, and excessive intraoperative blood loss.

**Supplementary Information:**

The online version contains supplementary material available at 10.1007/s11701-021-01192-w.

## Introduction

Prostate cancer (PCa) is the most commonly diagnosed tumor and the second leading cause of death from cancer in men [1]. Although several active treatments are included for the management of clinically localized PCa, radical prostatectomy (RP) is one of the most commonly selected treatment options that can be delivered by the open (ORP) or robot-assisted (RARP) approach [2]. RP is commonly carried out with concomitant extended pelvic lymph node dissection (ePLND) when anatomical staging of loco-regional lymph nodes is required [2]. However, prostate cancer surgery exposes patients to the risk of post-operative complications which can be classified according to Clavien–Dindo (CD) system [3, 4]. Postoperative complications prolong the length of hospital stay (LOHS) and increase the risk of readmission, thus impacting patient’s wellness, physician workload, and department-costs [5, 6]. Predictors of postoperative complications may have a pivotal role in the planning of general activity and anticipating the costs to operate a urological unit [5]. The aim of this study is to investigate the risk factors associated with major postoperative complications after ORP or RARP in a tertiary referral center.

## Materials and methods

### Surgical technique and perioperative management

The study is retrospective with prospectively collected data. Each patient provided informed-signed consent for data collection. Five skilled and dedicated surgeons performed operations of radical prostatectomy (RP) by open (ORP) or robot-assisted (RARP) approach with or without extended pelvic lymph node dissection (ePLND).

The decision to perform RARP or ORP was made according to the surgeon expertise (robot-assisted or open surgery) and patient preference.

RARP, which was delivered by the da Vinci Robot System (Intuitive Surgical, Inc., Sunnyvale, CA, USA), was performed through the trans peritoneal approach with anterograde prostatic dissection [7]. ORP was performed according to the technique of Walsh [8]. In intermediate risk cases, the decision to perform an ePLND was based on Briganti nomogram showing a risk of lymph node invasion greater than 5% [9]. In low-risk patients, ePLND was performed based on risk factors of tumor upgrading and upstaging [10–12]. In both surgical procedures, lymph nodes were sampled according to an anatomical template including bilateral external iliac (extending proximally to the crossing of the ureter), obturator, internal iliac, Marcille’s, common iliac and Cloquet’s nodal stations. The external iliac LN group was dissected laterally to the genitofemoral nerve at the lateral edge of the internal iliac artery and vein from the node of Cloquet to the ureteric crossing of the internal iliac artery as previously reported [13, 14].

Five experienced surgeons performed RARP with a bladder-neck sparing technique [15]. One surgeon performed more than 500 RARPs prior to the initiation of patient enrolment and he performed 66% of the procedures included in this study. The other four surgeons had performed between 50 and 60 procedures prior to the initiation of patient enrolment.

Prophylaxis of deep venous thrombosis with low molecular weight heparin was performed in all cases who underwent ePLND and in patients with comorbid risk factors; moreover, prophylaxis was prolonged till postoperative day (POD) 28. In RARP cases, transurethral 16 French Foley bladder catheter was placed and removed on post-operative day (POD) 12 without cystography according to our standard internal protocol. After December 2017, our policy of placing a pelvic drain was discontinued according to our previous reported experience [16]. In ORP patients, a transurethral 16 French Foley bladder catheter was placed and removed on POD 12 with or without cystography according to the surgeon’s decision; moreover, a pelvic drain was always placed and removed on POD 2 or 3.

### Predictors of complications

Clinical, pathological, surgical, peri-operative, intra-operative parameters were identified as potential predictors of complications. Serum prostate specific antigen (PSA; ng/mL) was determined by radioimmunoassay. Age (years), body mass index (BMI; kg/m^2^), total prostate volume (TPV, mL) and biopsy positive cores (BPC; percentage) were calculated for each case. Tumors were graded according to the 2014 International Society of Urologic Pathology (ISUP) [17]. Surgical specimens analyzed after 2014 were classified using the new classification and the Gleason grading group system [17, 18]. Surgical specimens evaluated between 2013 and 2014 were retrospectively classified by our dedicated pathologist after specimen review. Biopsy grade group (BGG) cancers included ISUP grade group 1–5. In appropriate cases, pelvic lymph node staging (cN) was performed by axial imaging modalities. Enlarged pelvic nodes measuring more than one centimeter in diameter were staged as cN1 disease. Axial imaging and total bone scanning was utilized to investigate the metastatic status when appropriate. Patients were classified into risk groups and staged according to the EAU guidelines recommendations on PCA [19].

Perioperative surgical risk was evaluated by the American Society of Anesthesiologists score (ASA) score system. Operating time (OT) (measured in minutes), was calculated as the interval between the first skin incision and the last skin suture in both RARP and ORP procedures. Surgical procedures were classified according to the RARP and OPR approach; moreover, ePLND (performed or not performed) was coded separately in each case. Intraoperative estimated blood loss (EBL) was measured in milliliters (mL).

### Assessment of complications

Postoperative surgical complications were prospectively recorded in each patient’s electronic medical record, and retrospectively collected for the present study. The events of complications were graded according to the Clavien–Dindo score system respecting Martin’s criteria as recommended by EAU guidelines [3, 4]. Only patients that provided signed informed consent were followed for complications and hospital readmission (RAD) after discharge. Patients were evaluated according to an institutional protocol including patient’s re-evaluation scheduled at 30, 60, 90, and 180 days after discharge by outpatient visit or phone interview by physicians.

### Objectives and design of the study

The aim of the study is to evaluate factors associated with the risk of complications after RP by both open and robot-assisted approach with or without ePLND. The following outcomes were identified as follows: (1) overall postoperative complications: subjects with Clavien–Dindo System (CDS) one through five versus cases without any complication; (2) moderate to major postoperative complications: cases with CDS two through five versus zero up to one and (3) major postoperative complications: subjects with CDS three through five versus patients with CDS zero up to two.

### Statistical methods

Summary statistics of the patient population and associations of factors between groups were evaluated. Data on continuous variables were reported as medians with their respective interquartile ranges (IQR) and differences between groups were analyzed by the Mann–Whitney’s *U *test*.* Data on categorical variables were calculated as frequency with percentages and differences between groups were analyzed with Pearson’s chi-squared test or Fisher exact test as appropriate.

The evaluation of factors associated with the risk levels of postoperative complications was assessed by the logistic regression model. Univariate models were computed first. Selection of independent variables was performed by Wald’s forward regression method in evaluating multivariate models. The software used to run the analysis was IBM-SPSS version 26. All tests were two-sided with *p* < 0.05 considered to indicate statistical significance.

## Results

### Analysis of overall complications risk

Overall, 1062 patients were included, 891 patients underwent RARP, and 171 ORP. Demographics of the patient population and subgroups are reported in Table 1. Complications occurred in 310 patients (29.1% of cases). Extended PLND was performed in 651 patients (61.3%). Major complications occurred in 58 cases (5.5%). Statistics of postoperative complications according to the Clavien–Dindo system are detailed in Supplementary Table 1. Clinical factors associated with complications included age, tumor grade and stage, surgical approach, ePLND and blood loss. Specifically, patients who had postoperative complications were significantly older with aggressive tumors and more likely underwent ORP or ePLND with increased intraoperative blood loss. Moreover, LOHS was significantly longer in cases with complications.

**Table 1:** Factors associated with complications after radical prostatectomy

	Population	No complications	Postoperative complications (*)	*p *value
Number (%)	1062	752 (70.7)	310 (29.1)	
Clinical factors				
Age (years); median (IQR)	66 (61–70)	65 (60–69)	66 (61–70)	0.005
BMI (kg/m^2^); median (IQR)	25.9 (24–28.1)	26 (24–28)	26 (23.9–28.4)	0.810
PSA (ug/L); median (IQR)	6.5 (4.9–9)	6.5 (4.9–9)	6.5 (4.9–9.1)	0.606
PV (mL); median (IQR)	40 (30–50)	39.5 (30–50)	40 (30–51)	0.705
BPC (%); median (IQR)	29 (19–50)	29 (18–47)	30.5 (20–50)	0.238
cT1; *n* (%)	712 (67)	520 (69.1)	192 (61.9)	0.023
cT2-3; *n* (%)	350 (33)	232 (30.9)	118 (38.1)	
cN0; i (%)	1031 (97.1)	730 (97.1)	301 (97.1)	0.984
cN1; *n* (%)	31 (2.9)	22 (2.9)	9 (2.9)	
ISUP < 3; *n* (%)	766 (72.1)	559 (74.3)	207 (66.8)	0.012
ISUP > 2; *n* (%)	296 (27.9)	193 (25.7)	103 (33.2)	
EAU low risk; *n* (%)	312 (29.4)	247 (32.8)	65 (21)	< 0.0001
EAU intermediate risk; *n* (%)	549 (51.7)	376 (50)	173 (55.8)	
EAU high risk; *n* (%)	201 (18.9)	129 (17.2)	72 (23.8)	
Pathological factors				
ISUP < 3; *n* (%)	551 (51.9)	401 (53.3)	150 (48.4)	0.143
ISUP > 2; *n* (%)	511 (48.1)	351 (46.7)	160 (51.6)	
pT2; *n* (%)	797 (75)	576 (76,6)	221 (71.3)	0.139
pT3a; *n* (%)	116 (10,9)	80 (10.6)	36 (11.6)	
pT3b; *n* (%)	149 (14)	96 (12.8)	53 (17.1)	
Negative SM; *n* (%)	778 (73.3)	561 (74.6)	217 (70)	0.124
Positive SM; *n* (%)	284 (26.7)	191 (25.4)	93 (30)	
pNx; *n* (%)	412 (38.8)	323 (43)	89 (28.7)	< 0.0001
pN0; *n* (%)	562 (52.9)	375 (49.9)	187 (60.3)	
pN1; *n* (%)	88 (8.3)	54 (7.2)	34 (11)	
Peri-operative factors				
ASA < 3; *n* (%)	962 (90.4)	684 (91)	278 (89.7)	0.516
ASA > 2; *n* (%)	100 (9.4)	68 (9)	32 (10.3)	
RARP; *n* (%)	891 (83.9)	667 (88.7)	224 (72.3)	< 0.0001
ORP; *n* (%)	171 (16.1)	85 (11.3)	86 (27.7)	
No ePLND; *n* (%)	411 (38.7)	322 (42.8)	89 (28.7)	< 0.0001
ePLND; *n* (%)	651 (61.3)	430 (57.2)	221 (71.3)	
Removed lymph nodes; median (IQR)	25 (18–32)	25 (19–32)	25 (17–31.5)	0.531
OT (min); median (IQR)	200 (165–238)	200 (165–237.7)	200 (161–240)	0.892
EBL (mL); median (IQR)	350 (200–600)	350 (200–500)	400 (200–600)	0.001
LOHS (days); median (IQR)	5 (4–6)	4 (4–5)	6 (4–8)	< 0.0001

*BMI* body mass index; *PSA* prostate specific antigen; *PV* prostate volume; *cT* tumor clinical stage; *cN* clinical nodal stage; *EAU* European Society of Urology; *ASA* American Society of Anesthesiologists Classification (ASA class); *ISUP* International Society of Urologic Pathology prostate cancer grade group system; *pT* pathological tumor stage; *pN* pathologic nodal stage; SM, surgical margin; *ePLND* extended pelvic lymph node dissection; *LOHS* length of hospital stay; (*) Clavien–Dindo score 1 through 5

The evaluation of clinical factors associated with the grades postoperative complications according to Clavien–Dindo classification is reported in Supplementary Table 2 where univariate and multivariate models are included. On multivariate analysis, the risk of overall postoperative complications were associated with ORP (odds ratio, OR = 2.038; *p* < 0.0001), ePLND (OR = 1.584; *p* < 0.0001) and EBL (OR = 1.001; *p* < 0.003); when ORP was removed, age (OR = 1.025; *p* < 0.029), ePLND (OR = 1.780; *p* < 0.0001) and EBL (OR = 1.001; *p* < 0.0001) were the independent predictors. The risk of postoperative complications greater than one compared to cases without or less than two, was predicted by ORP (OR = 2.976; *p* < 0.0001), ePLND (OR = 1.861; *p* < 0.0001) and EBL (OR = 1.001; *p* < 0.0001); when ORP was removed from the model, ePLND (OR = 2.519; *p* < 0.0001) and EBL (OR = 1.002; *p* < 0.0001) were the independent predictors of such risk. Major complications (CDS > 2) compared to cases without or less than three, were predicted only by ORP OR = 8.283; *p* < 0.0001); however, when ORP was removed from the model, ePLND (OR = 3.382; *p* = 0.001) and EBL (OR = 1.001; *p* < 0.0001) were found to be independent predictors.

- Risk models predicting major postoperative complications

Adjusted risk models predicting Clavien–Dindo postoperative complications are reported in Table 2. Independent exposure variables associated with the risk of postoperative complications included surgical approach, staging of loco-regional lymph nodes and intraoperative EBL above the third quartile. Exposure variables were evaluated for overall postoperative complications (Clavien–Dindo score 1 through 5a versus no complications), for moderate to major complications (Clavien–Dindo score above one versus zero up to one) and for major postoperative complications (Clavien–Dindo score above two versus none up to grade 2 complications). The results for overall and mild to major postoperative complications are detailed in Table 2. The evaluation of the risk model for major Clavien–Dindo complications was as follows. Considering the type of surgical approach, ORP compared to RARP increased the risk of major Clavien–Dindo complications from 2.8 to 19.3% (OR = 8.283; 95%CI 4.780–14.355). Evaluating surgical staging of pelvic lymph nodes, ePLND when compared to cases without anatomical staging increased the risk of major postoperative complications from 2.4 to 7.4% (OR = 3.090; 95%CI 1.543–6.191). Assessing intraoperative EBL, the risk of major postoperative complications was increased by EBL above the third quartile when compared to subjects with intraoperative EBL up to the third quartile (10.2% vs. 4.6%; OR 2.239; 95%, CI 1.233–4.064). The association of the exposure variables with the risk of postoperative complications was specifically stronger for major than for overall as well as for moderate through major Clavien–Dindo complications, as shown by the odds ratio values that are detailed in Table 2.

**Table 2:** Factors associated with the risk of complications after prostate cancer surgery according to the Clavien-Dindo System (CDS) in 1062 cases (multivariate models)

Exposure variable	Radical prostatectomy		Extended pelvic lymph node dissection		Estimated blood lost above the third quartile	
Level of exposure	RARP	ORP	No	Yes	No	Yes
CDS > 0 vs. CDS < 1						
CDS < 1; *n* (%)	667 (74.9)	85 (49.7)	322 (78.3)	430 (66.1)	654 (73.1)	98 (58.7)
CDS > 0; *n* (%)	224 (25.1)	86 (50.3)	89 (21.7)	221 (33.9)	241 (26.9)	69 (41.3)
OR (95%CI)	1	2,369 (1647–3.407) (*)	1	1,528 (1133–2060) (**)	1	1462 (1013–2108) (^)
*p* value	< 0.0001		0.005		0.042	
CDS > 1 vs. CDS < 2						
CDS < 2; *n* (%)	804 (90.2)	111 (64.9)	378 (92)	537 (82.5)	790 (88.3)	125 (74.9)
CDS ≥ 2; *n* (%)	87 (9.8)	60 (35.1)	33 (8)	114 (17.5)	105 (11.7)	42 (25.1)
OR (95%CI)	1	3710 (2431–5662) (*)	1	1694 (1096–2617) (**)	1	1633 (1046–2548) (^)
*p* value	< 0.0001		0.002	0.018	0.031	
CDS > 2 vs CDS < 3						
CDS < 3; *n* (%)	866 (97.2)	138 (80.7)	401 (97.6)	603 (92.6)	854 (95.4)	150 (89.8)
CDS ≥ 3; *n* (%)	25 (2.8)	33 (19.3)	10 (2.4)	48 (7.4)	41 (4.6)	17 (10.2)
OR (95%CI)	1	8283 (4780–14,355)	1	3090 (1543–6191) (***)	1	2239 (1233–4064) (^^)
*p* value	< 0.0001		0.001		0.008	

*OR* odds ratio; *CI* confidence interval; *RARP* robot-assisted radical prostatectomy; *ORP* open radical prostatectomy; (*), OR adjusted for extended pelvic lymph node dissection and blood lost above the third quartile; (**), OR adjusted for ORP and blood lost above the third quartile; (***), OR adjusted for blood lost above the third quartile; (^), adjusted for ORP and ePLND; (^^), adjusted for ePLND

The cohort has been further stratified in subgroups with or without ePLND, by surgical approach and blood loss up to the third quartile versus above. Distributions by subgroups are reported in Supplementary Table 3 and associations with the risk of Clavien–Dindo complications (univariate and multivariate analysis) are described in Table 3. Considering subgroups with and without ePLND, ORP referred to RARP independently associated with minor and major Clavien–Dindo complications when compared with both subjects without as well as grade 1–2 complications. Further details are illustrated in Table 3.

**Table 3:** Associations of type surgical approach and amount of blood lost with Clavien–Dindo complications after prostate cancer surgery by subgroups stratifying cases without and with extended pelvic lymph node dissection

	Univariate analysis			Multivariate analysis		
Subgroups	CDS 1–2 vs. CDS = 0	CDS > 2 vs. CDS = 0	CDS > 2 vs. CDS 1–2	CDS 1–2 vs. CDS = 0	CDS > 2 vs. CDS = 0	CDS > 2 vs. CDS 1–2
No ePLND	OR (95% CI)	OR (95% CI)	OR (95% CI)	OR (95% CI)	OR (95% CI)	OR (95% CI)
RARP	1	1	1	1	1	1
ORP	3699 (1207—11333)	19,286 (4110–90,505)	5214 (1065–25,522)	3139 (1002–9840)	18,339 (3742–89,874)	5214 (1065–25,522)
*p* value	0.022	< 0.0001	0.042	0.048	< 0.0001	0.042
BL ≤ 600 mL	1	1	1	1	1	1
BL > 600 mL	2178 (1150–4126)	1986 (0.406–9718)	0.912 (0.177–4699)	2004 (1045–3844)	1270 (0.231–6982)	0.634 (0.112–3598)
*p* value	0.017	0.397	0.912	0.036	0.783	0.607
ePLND	OR (95% CI)	OR (95% CI)	OR (95% CI)	OR (95% CI)	OR (95% CI)	OR (95% CI)
RARP	1	1	1	1	1	1
ORP	1683 (1111––2550)	7521 (3991–14,175)	4468 (2278–8763)	1506 (1010–2428)	6964 (3568–13,512)	4446 (2179–9072)
*p* value	0.014	< 0.0001	< 0.0001	0.045	< 0.0001	< 0.0001
BL ≤ 600 mL	1	1	1	1	1	1
BL > 600 mL	1505 (0.952–2380)	2698 (1385–5257)	1792 (0.877–3661)	1298 (0.792–2093)	1308 (0.632–2709)	1016 (0.471—2193)
*p* value	0.080	0.004	0.109	0.308	0.469	0.967

*OR* odds ratio; *CI* confidence interval; *RARP* robot-assisted radical prostatectomy; *ORP* open radical prostatectomy; *ePLND* extended pelvic lymph node dissection; BL, blood lost

## Discussion

Radical prostatectomy, and specifically RARP, has become the preferred PCa surgical treatment in western countries [2]. It is associated with major and minor complications that can occur over the short- and long-term after hospital discharge.

Complications after PCa surgery results in increased patient stress, physician workload and overall health cost [5].

There are few large studies dealing with complications after PCa surgery by both ORP and RARP with or without PLND. Pompe et al. showed that overall complications following PCa surgery by both ORP and RARP occurred in 1267 out of 4973 cases (25.6%) with PLND performed in 89.5% of cases; moreover, RARP was associated with less blood loss, shorter catheterization time, and lower risk of Grade II and III complications [20]. An Australian randomized study showed overall complications in only 6% of cases; however, in that study, a (limited) pelvic lymph node dissection was performed in only 36% of subjects [21]. A Sweden prospective study reported postoperative complication rates of 13.7% in 3706 patients with pelvic lymph node dissection being performed in only 448 cases (12.1%) [22]. In the present study, overall postoperative complications occurred in 29% of cases with ePLND being performed in 61.3% of the subjects. Complication rates of our study were closer to those reported by Pompe than to the Australian or Sweden studies [19–21]. They reported a lower Clavien–Dindo grade 1 complication rate (10.8% versus 15.1%), while complication rates for other the grades substantially overlapped [20].

In this context, it appears appropriate that our results, in the setting of a tertiary referral center where PCa surgery was performed with extended PLND in more than 60% of cases, show an overall complication rate of 29%, and major Clavien–Dindo complication rate of only 5.5%.

We have shown that ORP is an independent factor which predicts high grade complication, and high-grade complications are known to be associated with hospital readmission [23, 24]. Our findings are supported by the results from multiple multi-center studies [18, 19, 23] and might be explained by lower inflammatory response after robotic surgery compared to ORP [25]. Furthermore, surgery triggers the release of cortisol due to sympathetic activation. This induces a negative feedback mechanism that attempts to recover from the stress and this recovery may be faster in RARP cases [26, 27].

Our study also showed that ePLND and excessive intraoperative EBL were independently associated with the risk of minor through moderate and up to severe Clavien–Dindo postoperative complications.

Excessive intraoperative EBL might be related to dissection of complex tumors when also ePLND is performed. Locally advanced cancers do not have a well-defined plane of dissention and hemostasis is more difficult to maintain; additionally, large tumors invading the prostate capsule are associated with a complex vascular network because of angiogenetic factors; as such, dissection and bleeding control becomes more challenging, especially in cancers located at the apex where the neuro-vascular bundle is more represented [22].

When PLND is performed during RP, especially when an extended template is adopted, complications may occur not only because of uncontrolled clamping of the lymphatic vessels but also because of small bleeding vessels originating from the muscular pelvic wall of the obturator fossa. Anatomic human variability of the vessels and the lymphatic pelvic system is also a factor that should considered during ePLND, as shown by anatomical studies investigating this subject [28]. As such, an ePLND may result in complicated lymphoceles or pelvic hematomas requiring subsequent invasive procedures. Recently, Oderda et al., showed a PLND-related complication rate of 8.9% in a large cohort of patients (*n* = 14,921 at eight European tertiary referral centers after RP and ePLND). Interestingly, it was higher in patients with pN1 (8.5% vs. 12.6, *p* < 0.001) [29].

Considering clinical T2-3 PCa patients, a challenging prostate dissection may be complicated by excessive and uncontrolled bleeding from the prostate bed and from the small pelvic vessels. As a consequence, all these intraoperative issues result in the two main complications that occur during RP: pelvic hematomas and lymphoceles. Furthermore, when aggressive cancers of the mid-to-base regions of the prostate involve the seminal vesicles, the lymphatic network originating from the caudal and posterior parts of the gland are also involved resulting in multiple as well as and bilateral lymph node invasion [30, 31].

As such, the dissection of the posterior plane in higher stage PCa and ePLND become more challenging, because the seminal vesicles may be adherent to the posterior layer of Denovillier’s fascia and the pattern of the posterior lymphatic system network is altered [32]. This might result in greater Clavien–Dindo 3a-3b complications rates, is associated with procedures related to bleeding (pelvic hematomas) and complicated pelvic lymph lymphoceles. In our study, although pelvic hematomas and lymphoceles were the most frequent complications (8.8%), only 4.2% required invasive procedures for a complete resolution (Clavien–Dindo 3a or 3b). On the other hand, many physical factors should be considered when surgical approach is discussed with PCa patients including BMI which is related to high risk of Clavien-Dindo 3 and higher complications as well as perioperative and oncological outcomes [33, 34].

Our study results have important implications in clinical practice. Patients who undergo prostate cancer surgery should be counselled on the risks and severity of postoperative complications. Surgeons can reduce the risk of postoperative complications by working on risk factors at time of surgery. Reducing the risk of postoperative complications has implications on LOHS and costs. Moderate complications as well as major Clavien–Dindo complications may require referral to a tertiary center where these complications can be safely managed. Indeed, grade 2 complications include mainly pelvic hematomas that occur during hospital stay and require close monitoring and possible radiologic intervention.

Our study has some limitations. First, although data were collected prospectively, the analysis was retrospective and, as such, suffers the limits from these kinds of studies. Second, it was not a controlled study with the bias of selection of patients.

Although our study has limits, it also has much strength. First, the data were prospectively collected. Second, it was a large single center trial where both procedures are performed by skilled and experienced surgeons. Third, it is the first study analyzing specifically the risk of major postoperative and factors predicting such risk.

## Conclusions

In the present cohort, radical prostatectomy showed major postoperative complications that were independently predicted by the open approach, extended pelvic lymph-node dissection and excessive intraoperative blood loss.

Patients undergoing radical prostatectomy should be informed about the risk of post-operative complications during pre-operative counseling.

## Supplementary Information

Below is the link to the electronic supplementary material.

Supplementary file1 (XLS 25 KB)

Supplementary file2 (XLS 31 KB)

Supplementary file3 (XLSX 10 KB)

## References

[1] Siegel RL, Miller KD, Jemal A (2020). Cancer statistics, 2020. Cancer J Clin.

[2] Oberlin DT (2016). The effect of minimally invasive prostatectomy on practice patterns of American urologists. Urologic oncology seminars and original investigations.

[3] Dindo D, Demartines N, Clavien P-A (2004). Classification of surgical complications: a new proposal with evaluation in a cohort of 6336 patients and results of a survey. Ann Surg.

[4] Mitropoulos D (2013). Reporting and grading of complications after urologic surgical procedures: an ad hoc EAU guidelines panel assessment and recommendations. ActasUrol Esp.

[5] Gan ZS (2020). Correlation of relative value units with surgical complexity and physician workload in urology. Urology.

[6] Sebben M (2020). Open approach, extended pelvic lymph node dissection, and seminal vesicle invasion are independent predictors of hospital readmission after prostate cancer surgery: a large retrospective study. Minerva UrolNefrol.

[7] Menon M, Tewari A, Peabody J (2003). Vattikuti Institute prostatectomy: technique. J Urol.

[8] Walsh PC (1998). Anatomic radical prostatectomy: evolution of the surgical technique. J Urol.

[9] Briganti A (2012). Updated nomogram predicting lymph node invasion in patients with prostate cancer undergoing extended pelvic lymph node dissection: the essential importance of percentage of positive cores. EurUrol.

[10] Porcaro AB (2018). Clinical factors stratifying the risk of tumor upgrading to high-grade disease in low-risk prostate cancer. Tumori J.

[11] Porcaro AB (2016). Low-risk prostate cancer and tumor upgrading to higher patterns in the surgical specimen. Analysis of clinical factors predicting tumor upgrading to higher Gleason patterns in a contemporary series of patients who have been evaluated according to the modified Gleason score grading system. Urol Int.

[12] Porcaro AB (2017). Clinical factors of disease reclassification or progression in a contemporary cohort of prostate cancer patients elected to active surveillance. Urol Int.

[13] Porcaro AB (2019). Lymph nodes invasion of marcille’s fossa associates with high metastatic load in prostate cancer patients undergoing extended pelvic lymph node dissection: the role of “Marcillectomy”. Urol Int.

[14] Cacciamani GE (2019). Extended pelvic lymphadenectomy for prostate cancer: should the Cloquet's nodes dissection be considered only an option?. Minerva UrolNefrol.

[15] Freire MP (2009). Anatomic bladder neck preservation during robotic-assisted laparoscopic radical prostatectomy: description of technique and outcomes. EurUrol.

[16] Porcaro AB (2019). Is a drain needed after robotic radical prostatectomy with or without pelvic lymph node dissection? Results of a single-center randomized clinical trial. J Endourol.

[17] Epstein JI (2016). The 2014 International Society of Urological Pathology (ISUP) consensus conference on Gleason grading of prostatic carcinoma. Am J SurgPathol.

[18] Pierorazio PM (2013). Prognostic G leason grade grouping: data based on the modified G leason scoring system. BJU Int.

[19] Mottet N (2017). EAU-ESTRO-SIOG guidelines on prostate cancer. Part 1: screening, diagnosis, and local treatment with curative intent. EurUrol.

[20] Pompe RS (2018). Postoperative complications of contemporary open and robot-assisted laparoscopic radical prostatectomy using standardised reporting systems. BJU Int.

[21] Yaxley JW (2016). Robot-assisted laparoscopic prostatectomy versus open radical retropubic prostatectomy: early outcomes from a randomised controlled phase 3 study. Lancet.

[22] Wallerstedt Lantz A (2019). 90-Day readmission after radical prostatectomy: a prospective comparison between robot-assisted and open surgery. Scand J Urol.

[23] Moschini M (2017). Incidence and predictors of 30-day readmission after robot-assisted radical prostatectomy. ClinGenitourin Cancer.

[24] Xia L (2017). Predischarge predictors of readmissions and postdischarge complications in robot-assisted radical prostatectomy. J Endourol.

[25] Fracalanza S (2008). Is robotically assisted laparoscopic radical prostatectomy less invasive than retropubic radical prostatectomy? Results from a prospective, unrandomized, comparative study. BJU Int.

[26] Porcaro AB (2016). Robotic assisted radical prostatectomy accelerates postoperative stress recovery: Final results of a contemporary prospective study assessing pathophysiology of cortisol peri-operative kinetics in prostate cancer surgery. Asian J Urol.

[27] Porcaro AB (2015). Robotic-assisted radical prostatectomy is less stressful than the open approach: results of a contemporary prospective study evaluating pathophysiology of cortisol stress-related kinetics in prostate cancer surgery. J Robot Surg.

[28] Boscolo-Berto R (2020). The underestimated posterior lymphatic drainage of the prostate: an historical overview and preliminary anatomical study on cadaver. Prostate.

[29] Oderda M (2020). Indications for and complications of pelvic lymph node dissection in prostate cancer: accuracy of available nomograms for the prediction of lymph node invasion. BJU Int.

[30] Gil-Vernet JM (1996). Prostate cancer: anatomical and surgical considerations. Br J Urol.

[31] Porcaro AB (2017). Bilateral lymph node micrometastases and seminal vesicle invasion associated with same clinical predictors in localized prostate cancer. Tumori.

[32] Montorsi F (2012). Best practices in robot-assisted radical prostatectomy: recommendations of the Pasadena Consensus Panel. EurUrol.

[33] Porcaro AB (2020). High body mass index predicts multiple prostate cancer lymph node metastases after radical prostatectomy and extended pelvic lymph node dissection. Asian J Androl.

[34] Porcaro AB (2019). Body mass index is an independent predictor of Clavien-Dindo grade 3 complications in patients undergoing robot assisted radical prostatectomy with extensive pelvic lymph node dissection. J Robot Surg.

